# DEVELOPMENT AND VALIDATION OF PERSON-CENTERED MEALTIME CARE KNOWLEDGE AND SELF-EFFICACY ASSESSMENTS

**DOI:** 10.1093/geroni/igad104.1947

**Published:** 2023-12-21

**Authors:** Wen Liu, Heather Suh, Kyu Ri Lee, Cameron Coates, Kathleen Buckwalter

**Affiliations:** The University Of Iowa, Iowa City, Iowa, United States; The University of Iowa, Iowa City, Iowa, United States; The University of Iowa, Iowa City, Iowa, United States; The University of Iowa, Iowa City, Iowa, United States; The University of Iowa, Iowa City, Iowa, United States

## Abstract

Person-centered mealtime care is individually tailored and directly delivered care that engages individuals in mealtime activities and addresses their needs and preferences. Person-centered mealtime care is associated with positive mealtime behaviors and outcomes in older adults with dementia. Existing tools that assess caregiver knowledge and self-efficacy of mealtime care have low psychometric quality, and few tools focus on person-centered mealtime care. This study developed and tested the Person-Centered Mealtime Care Knowledge and Self-Efficacy Assessments. The 20-item Knowledge Assessment (total score = 0-20, higher score = better knowledge) and 15-item Self-Efficacy Assessment (items are scored from 1 = strongly disagree to 5 = strongly agree; total score = 15 - 75; higher score = better self-efficacy) were developed based on literature and expert reviews and finalized based on content validity. Seventy-two nursing home direct care staff (n=72, age = 45±12.1 years; 8.5% male; 12.3% non-white; 93% provided dementia mealtime care; 89% experienced challenges during mealtime) completed the assessments. The two assessments showed adequate content validity (Scale-CVI = 0.95-1.00; item-CVI = 0.83-1.00) and acceptable-to-good internal consistency (Cronbach’s Alpha = .651 and .984 for Knowledge and Self-Efficacy Assessments, respectively). There was a weak and significant correlation between Knowledge and Self-Efficacy Assessments (rs = .262, p = .026). Findings provide preliminary psychometric evidence of the two assessments to measure caregivers’ knowledge and self-efficacy in providing person-centered mealtime care. Future validity testing and item-level testing using Item Response Theory is needed among larger populations of diverse caregivers to accumulate rigorous psychometric evidence.

